# Early Fibrinogen‐to‐Albumin Ratio and Poor Functional Outcome After Acute Ischemic Stroke: A Systematic Review and Meta‐Analysis

**DOI:** 10.1002/brb3.71929

**Published:** 2026-11-13

**Authors:** Qin Yang, Wenke Li, Yiting Deng, Jiawei Meng, Anling Luo, Yujia Yang, Xuzi Li, MuKe Zhou

**Affiliations:** ^1^ Department of Neurology West China Hospital Sichuan University Chengdu Sichuan China; ^2^ Department of Head and Neck Oncology West China Hospital Sichuan University Chengdu Sichuan China; ^3^ Center of Cerebrovascular Diseases West China Hospital Sichuan University Chengdu Sichuan China

**Keywords:** acute ischemic stroke, fibrinogen‐to‐albumin ratio, functional outcome, meta‐analysis

## Abstract

**Background**: Early fibrinogen‐to‐albumin ratio (FAR) has been linked to functional outcome after acute ischemic stroke, but reported exposure definitions and numerical scales vary. We assessed whether higher early FAR was associated with poor functional outcome.

**Methods**: We searched five databases through July 23, 2026 for adult acute ischemic stroke studies that measured FAR during initial assessment or before reperfusion and reported adjusted odds ratios (ORs) for modified Rankin Scale outcomes. Categorical and continuous estimates were pooled separately using restricted maximum likelihood random‐effects models with modified Hartung–Knapp confidence intervals (CIs). Continuous effects were harmonized to a common FAR_100 scale. We performed leave‐one‐out, scale‐restriction, and reperfusion analyses and assessed risk of bias with Quality In Prognosis Studies (QUIPS). The review was registered in PROSPERO (CRD420261459955).

**Results**: Twelve studies included 13,253 participants; 12,579 contributed effect estimates. Study‐defined higher FAR was associated with poor outcome (five studies; 10,968 participants; OR, 2.32; 95% CI: 1.37–3.91; *I* ^2^ = 82.7%). Across seven continuous studies (1611 participants), each 1‐unit increase in FAR_100 was associated with poor outcome (OR, 1.30; 95% CI: 1.14–1.48; *I* ^2^ = 51.1%). Leave‐one‐out CIs remained above 1. Excluding studies requiring scale conversion produced a similar estimate (five studies; 1173 participants; OR, 1.29; 95% CI: 1.16–1.45; *I* ^2^ = 10.5%).

**Conclusions**: Higher early FAR was associated with poor functional outcome in both categorical and continuous analyses. Prospective studies should test whether FAR adds prognostic information to established stroke models. Available evidence came from observational studies.

## Graphical abstract

Across 12 studies, higher early fibrinogen‐to‐albumin ratio was associated with poor functional outcome after acute ischemic stroke in both categorical (OR 2.32) and continuous (OR 1.30 per FAR_100 unit) analyses. Continuous estimates remained stable in leave‐one‐out and scale‐restriction analyses.

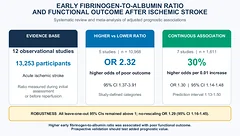

## 1 Introduction

Acute ischemic stroke remains a major cause of death and long‐term disability worldwide (GBD 2021 Stroke Risk Factor Collaborators 2024). Functional recovery varies substantially, even among patients who receive similar acute treatment. Early prognostic information helps clinicians plan monitoring, rehabilitation, and follow‐up. However, commonly used clinical variables do not fully explain why outcomes differ between patients. Routine blood tests may offer simple, reproducible prognostic markers because they are already available during initial assessment.

Fibrinogen contributes to clot formation, platelet aggregation, blood viscosity, and inflammatory signaling. Higher fibrinogen concentrations have been associated with poor functional outcome after ischemic stroke (Parikh et al. 2020; Barakzie et al. 2023; Hou et al. 2021). Albumin reflects nutritional reserve and is involved in antioxidant activity and endothelial homeostasis. Lower albumin concentrations have also been linked to poor functional outcomes after stroke (Parikh et al. 2020; Barakzie et al. 2023; Zhou et al. 2021). The fibrinogen‐to‐albumin ratio (FAR) combines these two routinely measured proteins. A high FAR may indicate increased procoagulant and inflammatory activity, reduced physiological reserve, or both. FAR has also been associated with hemorrhagic transformation after ischemic stroke (Ruan et al. 2021).

Several cohort studies have examined early FAR in relation to modified Rankin Scale (mRS) outcomes after acute ischemic stroke (Zheng et al. 2021; Zhai et al. 2022; Wang et al. 2023, 2024; Chen et al. 2024). Study populations included unselected ischemic stroke cohorts and patients with lacunar, pontine, or large‐artery atherosclerotic stroke. Other cohorts included patients treated with intravenous thrombolysis or mechanical thrombectomy. However, studies differed in how they defined and analyzed FAR. Some compared groups based on study‐specific thresholds or distributional quantiles, whereas others modeled FAR as a continuous variable. Sampling windows, numerical scales, follow‐up periods, and mRS thresholds also varied. A valid pooled analysis should therefore separate categorical from continuous estimates and convert continuous estimates to a common scale.

We conducted a systematic review and meta‐analysis to assess the association between early FAR and poor functional outcome after acute ischemic stroke. We analyzed study‐defined FAR categories separately from continuous FAR measurements. Before pooling, we converted continuous estimates to FAR_100, defined as fibrinogen (g/L) divided by albumin (g/L) and multiplied by 100. Additional analyses examined whether results changed after removing individual studies, excluding studies requiring mathematical scale conversion, or restricting the dataset to reperfusion‐treated cohorts.

## 2 Materials and Methods

### 2.1 Study Design and Reporting

This review followed PRISMA 2020 and MOOSE recommendations and was registered in PROSPERO (CRD420261459955) (Page et al. 2021; Stroup et al. 2000). Two reviewers independently screened records, assessed full texts, extracted data, and evaluated risk of bias; disagreements were resolved by consensus. Further details are provided in Sections S1–S5.

### 2.2 Eligibility Criteria

We included observational studies of adults with imaging‐confirmed acute ischemic stroke. FAR had to be measured during initial assessment or before reperfusion. Eligible reports provided an adjusted odds ratio (OR) with a 95% confidence interval (CI) for poor functional outcome based on a dichotomized‐mRS between 30 days and 1 year. Posttreatment‐only FAR and reports without an eligible FAR‐specific adjusted estimate were excluded.

### 2.3 Information Sources and Search Strategy

We searched PubMed/MEDLINE, Embase, Web of Science Core Collection, Scopus, and Wanfang from inception through July 23, 2026, supplemented by public discovery sources and citation searching. Complete strategies and database yields are provided in the Supplementary Search Strategies file and Table S3, respectively.

### 2.4 Study Selection and Data Extraction

Two reviewers independently selected studies and extracted data. We retained one estimate per cohort within each panel, prioritizing the primary follow‐up and most fully adjusted eligible model. Categorical and continuous FAR effects were analyzed separately.

### 2.5 Effect Measures and Scale Harmonization

Categorical analyses compared study‐defined higher and lower FAR groups. Continuous effects were harmonized to FAR_100 on the log‐OR scale. When the reported regression variable was *c* × FAR_100 and the OR was per 1‐unit increase in that variable, the log OR and its standard error were multiplied by *c*. For Lai et al. (2024) and Zhang et al. (2024), *c* = 10 was inferred from reported units and/or FAR values. Zhang reported entering continuous predictors at their original values; its FAR measurement units were not explicit. Study‐specific evidence, reported or assumed regression increments, and conversion examples are provided in Section S3 and Table S2.

### 2.6 Risk of Bias

Two reviewers independently rated the six Quality In Prognosis Studies (QUIPS) domains as low, moderate, or high risk of bias (Hayden et al. 2013).

### 2.7 Statistical Analysis

We pooled log ORs using random‐effects models with restricted maximum likelihood estimation of between‐study variance (*τ* ^2^) and modified Hartung–Knapp CIs (Veroniki et al. 2016; IntHout et al. 2014; Röver et al. 2015). Cochran's *Q* was evaluated against a chi‐square distribution with *k*−1 degrees of freedom, where *k* is the number of studies. Heterogeneity was quantified as *I* ^2^ = max{0, [*Q*−(*k*−1)]/*Q*} × 100%. We report pooled ORs, heterogeneity statistics, and prediction intervals; full numerical details are provided in Table S7. Robustness analyses used leave‐one‐out and two nested scale restrictions; reperfusion‐cohort and meta‐regression analyses were exploratory. Small‐study tests were not used because each primary panel contained fewer than 10 studies. Analyses used R 4.6.1 and metafor 5.0‐1.

### 2.8 Use of Artificial Intelligence

During the preparation of this work, the authors used OpenAI Codex to assist with Chinese‐to‐English translation, language editing, and manuscript formatting. The authors subsequently reviewed and edited all outputs, independently verified the data, analyses, references, and interpretations, and took full responsibility for the content of the article.

## 3 Results

### 3.1 Study Selection

The five databases yielded 178 records: 31 from PubMed/MEDLINE, 32 from Embase, 25 from Web of Science, 33 from Scopus, and 57 from Wanfang. The four international bibliographic databases supplied 121 records, of which 74 were duplicates, leaving 47 unique records. Adding the 57 Wanfang records produced 104 records for title and abstract screening. At this stage, we excluded 84 records and reviewed 20 full‐text reports. Eight reports were excluded because exposure timing was ineligible, follow‐up functional outcomes were absent, the design was diagnostic, or no FAR‐specific effect could be extracted. Twelve studies met the eligibility criteria and were included in the quantitative synthesis (Figure 1; Table S1).

**FIGURE 1:**
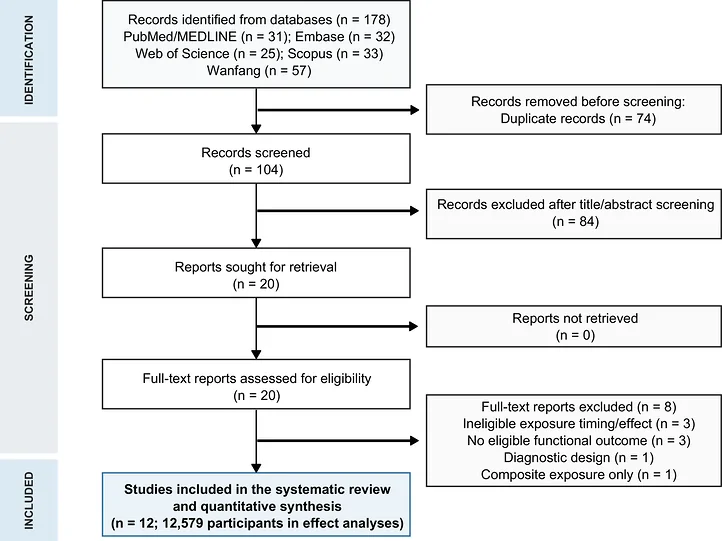
**Study selection**. The preferred reporting items for systematic reviews and meta‐analyses (PRISMA) 2020 flow diagram shows records identified, duplicates removed, reports assessed in full, and studies included in the systematic review and meta‐analysis.

### 3.2 Study Characteristics

The 12 included studies were published between 2021 and 2026 (Zheng et al. 2021; Zhai et al. 2022; Wang et al. 2023, 2024; Chen et al. 2024; Tang et al. 2024; Lai et al. 2024; Ozdogru et al. 2024; Zhang et al. 2024; Du et al. 2026; Wei et al. 2026; Meng et al. 2026). Eleven studies were conducted in China, and one study was conducted in Turkey. Across studies, 13,253 participants had baseline FAR data, and sample sizes ranged from 103 to 8984. A total of 12,579 participants contributed to the selected effect estimates. Study populations included unselected acute ischemic stroke, lacunar or pontine infarction, large artery atherosclerosis stroke, and patients receiving intravenous thrombolysis or endovascular therapy. Follow‐up ranged from 30 days to 1 year. Most studies defined poor functional outcome as mRS greater than 2 or mRS scores of 3–6. One study used mRS scores of 4–6 (Table 1).

**TABLE 1:** Characteristics of included studies.

Study	Country	Design and population	Baseline analyzable/effect‐analysis sample	FAR timing and scale	Exposure contrast	Functional outcome	Effect estimate	Analysis panel
Zheng et al. (2021)	China	Prospective registry secondary analysis; acute lacunar stroke	371/322	Within 48 h of admission; Fib (g/L)/Alb (g/L)	High ≥0.077 vs. low <0.077	3‐month mRS 3–6	aOR 2.20 (1.14–4.24)	Categorical
Zhai et al. (2022)	China	Prospective cohort; acute pontine infarction	264/264	Morning after admission; FAR_100	Per 1‐unit increase in FAR_100	3‐month mRS ≥3	aOR 1.293 (1.150–1.453)	Continuous
Wang et al. (2023)	China	Prospective cohort; large artery atherosclerosis stroke	809/809	Within 72 h of onset; Fib (g/L)/Alb (g/L)	Q4 >0.087 vs. Q1 <0.064	1‐year mRS 3–6	aOR 3.30 (1.57–6.94)	Categorical
Wang et al. (2024)	China	National multicenter prospective registry; CNSR‐III AIS	8984/8746	Baseline or within 7 days; reported numerical scale approximately 7–11	Q4 vs. Q1	1‐year mRS 3–6	aOR 1.64 (1.35–2.00)	Categorical
Chen et al. (2024)	China	Retrospective cohort; AIS treated with intravenous thrombolysis	591/204	Before thrombolysis; FAR_100	Per 1‐unit increase in FAR_100	3‐month mRS >2	aOR 1.19 (1.03–1.38)	Continuous
Ozdogru et al. (2024)	Turkey	Retrospective cohort; AIS treated with mechanical thrombectomy	103/103	At admission; raw Fib (g/L)/Alb (g/L) analyzed as FAR x 100	Per 1‐unit increase in FAR_100	3‐month mRS >2	aOR 1.247 (1.036–1.501)	Continuous
Tang et al. (2024)	China	Retrospective cohort; older adults with AIS treated by endovascular therapy	376/376	Before the procedure; numerical scale consistent with FAR_100	Per 1‐unit increase in FAR_100	90‐day mRS >2	aOR 1.470 (1.130–1.911)	Continuous
Lai et al. (2024)	China	Retrospective cohort; large artery atherosclerosis infarction without reperfusion therapy	196/196	Within 24 h of admission; reported units imply 10 × FAR_100	Per 1‐unit increase in FAR_100	90‐day mRS >2	aOR 4.19 (1.06–16.43)	Continuous
Wei et al. (2026)	China	Retrospective cohort; successfully recanalized large vessel occlusion after thrombectomy	226/226	Before the procedure; FAR_100	Per 1‐unit increase in FAR_100	90‐day mRS ≥3	aOR 1.539 (1.221–1.941)	Continuous
Zhang et al. (2024)	China	Retrospective single‐center cohort; acute cerebral infarction admitted within 72 h	242/242	Within 24 h of admission; reported values suggest 10 × FAR_100	Per 1‐unit increase in FAR_100	3‐month mRS 3–6	aOR 51.80 (2.04–1317.21)	Continuous
Meng et al. (2026)	China	Retrospective cohort; AIS treated with endovascular thrombectomy	254/254	At admission; Fib (g/L)/Alb (g/L)	Q5 ≥0.099 vs. Q1 ≤0.058	30‐day mRS ≥3	aOR 1.519 (0.609–3.788)	Categorical
Du et al. (2026)	China	Retrospective cohort; anterior‐circulation large artery atherosclerosis infarction without reperfusion therapy	837/837	First admission blood sample; Fib (g/L)/Alb (g/L)	High >0.0908 vs. low ≤0.0908	3‐month mRS 4–6	aOR 3.372 (2.678–4.274)	Categorical

Abbreviations: FAR, fibrinogen‐to‐albumin ratio; mRS, modified Rankin Scale.

Five studies comprising 10,968 participants reported categorical FAR estimates based on binary thresholds, quartiles, or quintiles. Seven studies comprising 1611 participants reported continuous effects standardized to each 1‐unit increase in FAR_100.

### 3.3 Risk of Bias

Of the 72 domain‐level QUIPS judgments, 60 (83.3%) were low risk, 11 (15.3%) were moderate risk, and 1 (1.4%) was high risk. Five studies were rated low risk across all six domains. Prognostic factor measurement and outcome measurement were rated low risk in all 12 studies. Moderate‐risk judgments were most frequent in statistical analysis and reporting (six studies), followed by study confounding (three studies). The only high‐risk judgment concerned attrition in one study. Table S4 and Figure 2 present the final domain‐level consensus judgments.

**FIGURE 2:**
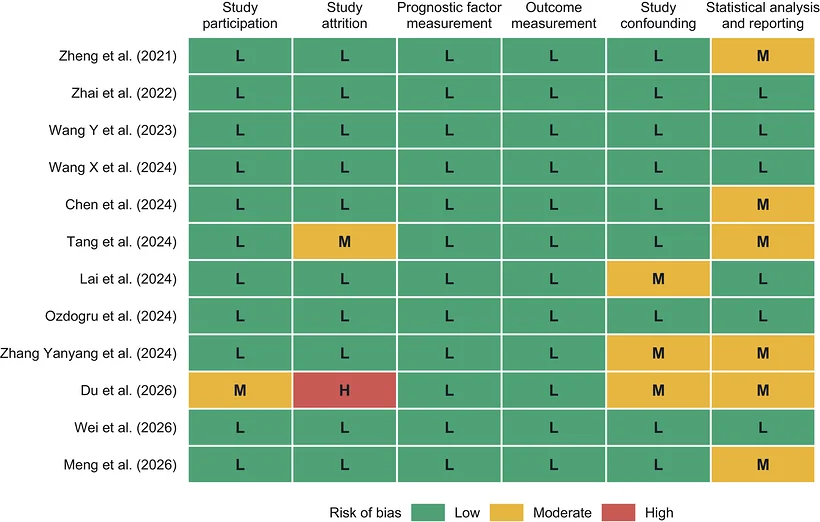
**Risk‐of‐bias assessment of the included studies using the Quality In Prognosis Studies (QUIPS) tool**. Cells show the final consensus judgments of two independent reviewers (low, moderate, or high risk of bias) for each study and domain.

### 3.4 Categorical FAR and Poor Functional Outcome

The categorical analysis included five studies and 10,968 participants in total. Study‐defined higher FAR was associated with poor functional outcome (pooled OR 2.32, 95% CI: 1.37–3.91; Figure 3A; Table 2). Between‐study heterogeneity was substantial (*τ* ^2^ = 0.108; *I* ^2^ = 82.7%; *Q* = 23.16, 4 degrees of freedom; *p* < 0.001). The 95% prediction interval ranged from 0.69 to 7.74 and crossed 1. All study‐specific point estimates were greater than 1 across the five studies.

**FIGURE 3:**
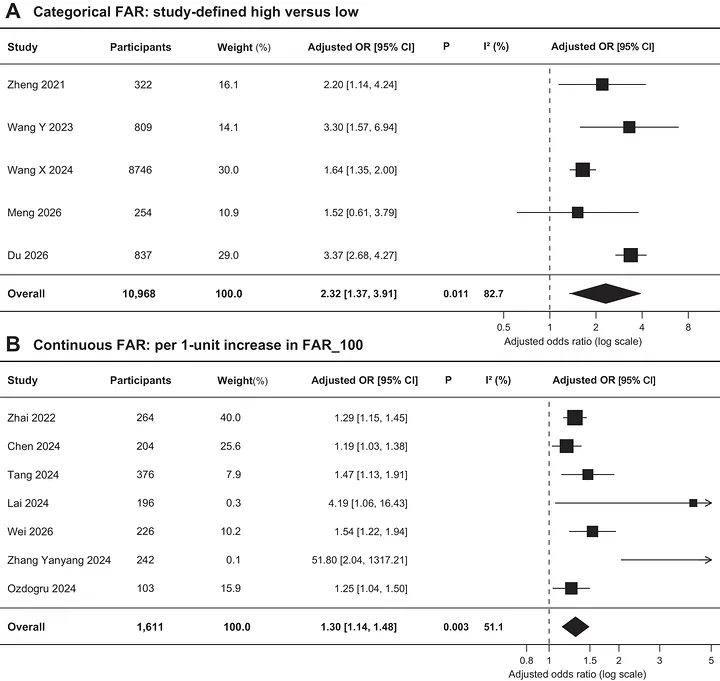
**Association between early fibrinogen‐to‐albumin ratio and poor functional outcome after acute ischemic stroke**. (A) Adjusted odds ratios comparing study‐defined high and low FAR categories. (B) Adjusted odds ratios per 1‐unit increase in FAR_100, defined as fibrinogen (g/L) divided by albumin (g/L) and multiplied by 100. The left table reports sample sizes, adjusted ORs with 95% CIs, random‐effects weights, pooled‐effect *p* values, and *I* ^2^. *p* values are from two‐sided modified Hartung–Knapp tests of the pooled null OR = 1; *I* ^2^ is *Q*‐based. Squares and horizontal lines show study‐specific estimates and 95% CIs; square size reflects the random‐effects weight. Diamonds show pooled estimates. Arrowheads indicate point estimates or confidence limits beyond the plotted axis. CI, confidence interval; FAR, fibrinogen‐to‐albumin ratio; OR, odds ratio.

**TABLE 2:** Summary of primary, robustness, and reperfusion‐treatment meta‐analyses.

Analysis	Exposure contrast	Studies	Participants	Pooled adjusted OR (95% CI)	*I* ^2^ (%)	95% PI
Categorical FAR (primary)	Study‐defined high or highest category vs. low or lowest category	5	10,968	2.32 (1.37–3.91)	82.7	0.69–7.74
Continuous FAR_100 (primary)	Per 1‐unit increase in FAR_100	7	1611	1.30 (1.14–1.48)	51.1	1.13–1.50
Continuous FAR_100 without mathematical rescaling (robustness)	Per 1‐unit increase in FAR_100	5	1173	1.29 (1.16–1.45)	10.5	Not calculated
Continuous FAR_100 with an explicit or confirmed scale (robustness)	Per 1‐unit increase in FAR_100	4	797	1.28 (1.12–1.47)	14.0	Not calculated
Continuous FAR_100 in reperfusion‐treated cohorts (exploratory)	Per 1‐unit increase in FAR_100	4	909	1.32 (1.08–1.60)	32.9	Not calculated

*Note*: All pooled estimates were obtained using restricted maximum likelihood random‐effects models with modified Hartung–Knapp 95% confidence intervals. Categorical and continuous FAR represent separate estimands and should not be compared directly. Continuous estimates are reported per 1‐unit increase in FAR_100, where FAR_100 is fibrinogen (g/L) divided by albumin (g/L) and multiplied by 100. The analysis without mathematical rescaling excluded Lai et al. (2024) and Zhang et al. (2024); the explicit or confirmed‐scale analysis additionally excluded Tang et al. (2024). Prediction intervals are shown only for the two primary analyses. Full model and heterogeneity statistics are provided in Table S7.

Abbreviations: CI, confidence interval; FAR, fibrinogen‐to‐albumin ratio; OR, odds ratio; PI, prediction interval.

### 3.5 Continuous FAR and Poor Functional Outcome

The continuous analysis included seven studies and 1611 participants in total. Each 1‐unit increase in FAR_100 was associated with higher odds of poor functional outcome (pooled OR 1.30, 95% CI: 1.14–1.48; Figure 3B; Table 2). The estimated between‐study variance was *τ* ^2^ < 0.001 (*I* ^2^ = 51.1%; *Q* = 12.27, 6 degrees of freedom; *p* = 0.056). The 95% prediction interval ranged from 1.13 to 1.50 across studies.

### 3.6 Robustness and Scale‐Restriction Analyses

Leave‐one‐out analyses showed that no single study determined either primary result. After omitting each categorical study in turn, pooled ORs ranged from 1.88 to 2.87. All recalculated 95% CIs remained above 1, and the smallest lower confidence limit was 1.11. Across the categorical leave‐one‐out analyses, *I* ^2^ values ranged from 20.5% to 87.0%. Omitting Wang et al. (2024) reduced categorical *I* ^2^ to 23.3%, whereas omitting Du et al. (2026) reduced it to 20.5%. For continuous FAR, leave‐one‐out pooled ORs ranged from 1.28 to 1.34 per 1‐unit increase in FAR_100. Every recalculated 95% CI remained above 1, and the smallest lower limit was 1.05. Across the continuous leave‐one‐out analyses, *I* ^2^ values ranged from 31.4% to 59.2% (Figure 4A,B; Table S5).

**FIGURE 4:**
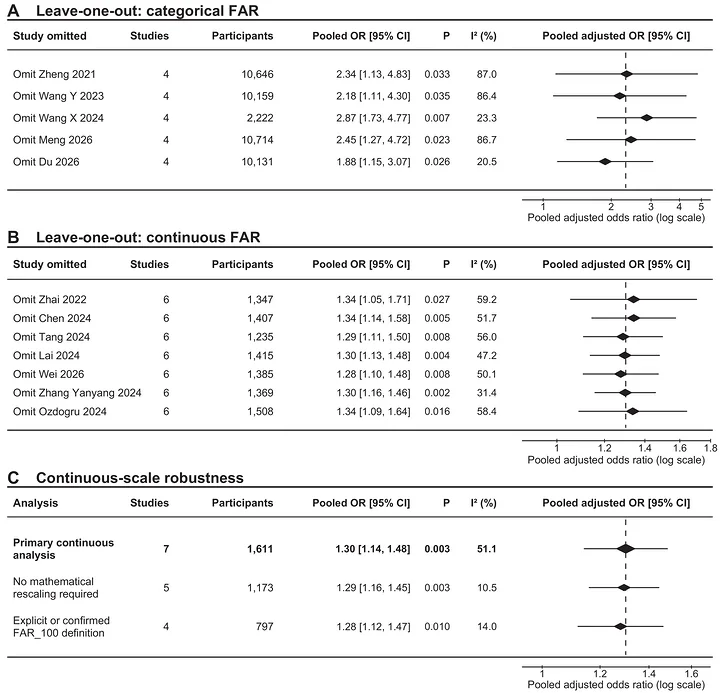
**Leave‐one‐out and continuous‐scale robustness analyses**. (A and B), Pooled adjusted odds ratios after omission of each study from the categorical and continuous analyses, respectively. (C) Primary continuous estimate compared with two nested scale‐restriction analyses. Each panel places a complete numerical table beside the forest plot. *p* values are from two‐sided modified Hartung–Knapp tests of the pooled null OR = 1; *I* ^2^ is *Q*‐based. Dashed vertical lines indicate the primary pooled estimate, and dotted vertical lines indicate the null value. Estimates were obtained using restricted maximum likelihood random‐effects models with modified Hartung–Knapp 95% confidence intervals. Continuous estimates are reported per 1‐unit increase in FAR_100, where FAR_100 is fibrinogen (g/L) divided by albumin (g/L) and multiplied by 100. CI, confidence interval; FAR, fibrinogen‐to‐albumin ratio; OR, odds ratio.

After excluding Lai et al. (2024) and Zhang et al. (2024), five studies with 1173 participants remained in the analysis. The pooled estimate was OR 1.29 (95% CI: 1.16–1.45; *I* ^2^ = 10.5%; *Q* = 4.47, 4 degrees of freedom; *p* = 0.346). These two excluded studies required conversion to the common FAR_100 scale. The stricter analysis retained four studies with an explicit or confirmed FAR_100 definition and included 797 participants. This analysis produced an OR of 1.28 (95% CI: 1.12–1.47; *I* ^2^ = 14.0%; *Q* = 3.49, 3 degrees of freedom; *p* = 0.322). Both restricted estimates were similar to the primary estimate from the continuous analysis (Figure 4C; Table 2; Table S6).

### 3.7 Exploratory Analysis in Reperfusion‐Treatment Cohorts

Four continuous‐effect studies included 909 patients treated with intravenous thrombolysis or endovascular therapy. In this subset, each 1‐unit increase in FAR_100 was associated with poor functional outcome (OR 1.32, 95% CI: 1.08–1.60). Between‐study heterogeneity was low‐to‐moderate (*τ* ^2^ = 0.0054; *I* ^2^ = 32.9%; *Q* = 4.47, 3 degrees of freedom; *p* = 0.215). The exploratory meta‐regression included all seven available studies. No interaction was detected. The ratio of ORs for other or unselected acute ischemic stroke cohorts versus reperfusion‐treatment cohorts was 1.02 (95% CI: 0.65–1.59; *F*[1,5] = 0.011; *p* for interaction = 0.920) (Table 2; Figure S1; Table S6).

## 4 Discussion

The pooled results showed that higher early FAR was associated with poor functional outcome after acute ischemic stroke. Study‐defined higher FAR corresponded to approximately 2.3‐fold greater odds, whereas each 1‐unit increase in FAR_100 corresponded to 30% greater odds. The two analyses address different clinical questions in this review. The categorical analysis compares the FAR groups defined within each study, whereas the continuous analysis quantifies change on a common scale. Categorical effects varied substantially, and the prediction interval crossed 1. This variation likely reflects differences in thresholds, grouping methods, and study populations. The association remained positive when Wang et al. (2024) or Du et al. (2026) was omitted, although heterogeneity decreased markedly. The categorical data show an association with study‐defined higher FAR but do not identify a single cutoff for clinical use.

After conversion to FAR_100, continuous effects were more directly comparable across studies. Both the pooled CI and prediction interval remained above 1. The pooled estimate changed little after individual studies or mathematically converted effects were excluded. Excluding converted effects gave an OR of 1.29 with *I* ^2^ of 10.5%, compared with 1.30 in the seven‐study primary analysis. Restricting the analysis to four studies with an explicit or confirmed FAR_100 definition produced a similar estimate. The consistent results make it unlikely that one influential study or the conversion method accounted for the association. However, the included studies still differed in sampling windows and laboratory reporting.

The reperfusion subset showed an association similar to the main continuous analysis. Its pooled OR was 1.32 per 1‐unit increase in FAR_100. Meta‐regression found no interaction by treatment setting, although the analysis included few studies and a heterogeneous comparison group. The association was therefore observed in reperfusion‐treated cohorts, but equal effects across treatment settings cannot be assumed. Individual patient data are needed to assess whether treatment type, recanalization status, or onset‐to‐sampling time changes the association.

Several biological pathways could explain the association, although none is specific to FAR. Fibrinogen rises during acute inflammation and contributes to fibrin formation, platelet aggregation, and blood viscosity (Parikh et al. 2020; Barakzie et al. 2023; Hou et al. 2021). Albumin may decline during systemic inflammation and also reflects nutritional reserve and antioxidant capacity (Parikh et al. 2020; Barakzie et al. 2023; Zhou et al. 2021). A higher FAR may therefore reflect greater thrombo‐inflammatory activity, lower physiological reserve, or both. Either process could affect infarct progression and recovery, but the included studies cannot identify the responsible mechanism. Reverse causation also remains possible because greater stroke severity may alter acute‐phase protein levels.

Fibrinogen and albumin are routinely measured, allowing FAR to be calculated without an additional assay. However, our results do not establish whether FAR improves prediction beyond age, baseline National Institutes of Health Stroke Scale score, imaging findings, and reperfusion status. Most included studies examined association rather than incremental prediction, and their covariate adjustments differed. FAR should not yet be used as a stand‐alone decision rule. External validation studies should prespecify the sampling window and FAR formula. They should then test calibration, discrimination, reclassification, and decision‐curve net benefit after adding FAR to an established clinical model.

The review searched four international databases and Wanfang and then retained every study meeting the prespecified criteria for the relevant primary analysis. We analyzed categorical and continuous effects separately and checked the reported units before converting continuous effects. Modified Hartung–Knapp CIs were used because each analysis included few studies. Leave‐one‐out and nested scale‐restriction analyses examined the influence of individual studies and scale conversion.

All included studies were observational, so the pooled ORs describe prognostic associations rather than causal effects. Eleven studies were conducted in China, which may limit generalizability to other populations. Stroke subtype, treatment setting, blood‐sampling time, follow‐up duration, and mRS threshold varied across studies. Two continuous effects required conversion to FAR_100, and one study described the scale less clearly than the strict subset. The stable restricted analyses support the main result but cannot replace standardized prospective measurement. Residual confounding remains possible because adjustment sets differed across studies. Some studies did not fully adjust for stroke severity, nutritional status, infection, or treatment‐related variables. We did not search the China National Knowledge Infrastructure, so some eligible Chinese‐language studies may have been missed. Each primary analysis also included fewer than 10 studies, preventing reliable tests for publication bias or small‐study effects.

Future studies should report FAR as fibrinogen (g/L) divided by albumin (g/L), analyze FAR_100 continuously, and prespecify any categorical threshold. Whenever possible, blood should be collected within a narrow interval before reperfusion treatment. Prognostic models should adjust for age, baseline stroke severity, premorbid function, major vascular risk factors, imaging severity, and treatment status. They should avoid adjustment for variables measured after FAR that may lie between the exposure and outcome. Future studies should test whether FAR improves an established prognostic model in independent cohorts. An interpretive summary is provided in Table S8.

## 5 Conclusions

Higher early FAR was associated with poor functional outcome after acute ischemic stroke in both categorical and continuous analyses. The continuous association remained stable after each study was omitted and after effects requiring conversion were excluded. FAR is an accessible candidate prognostic marker that warrants prospective evaluation using standardized measurement. Future work should determine whether FAR improves existing prediction models and identify clinically useful thresholds.

## Author Contributions

**Qin Yang**: conceptualization, methodology, formal analysis, investigation, data curation, software, visualization, writing – original draft. **Wenke Li**: methodology, formal analysis, investigation, data curation, validation, writing – original draft. **Yiting Deng**: investigation, data curation, validation, writing – review and editing. **Jiawei Meng**: investigation, data curation, validation, writing – review and editing. **Anling Luo**: methodology, validation, writing – review and editing. **Yujia Yang**: investigation, data curation, writing – review and editing. **Xuzi Li**: investigation, validation, writing – review and editing. **MuKe Zhou**: conceptualization, methodology, supervision, project administration, validation, funding acquisition, writing – review and editing. All authors read and approved the final manuscript and accept accountability for the work.

## Funding

This work was supported by the Noncommunicable Chronic Diseases‐National Science and Technology Major Project (grant number 2024ZD0527700).

## Ethics Statement

The authors have nothing to report.

## Conflicts of Interest

The authors declare no conflicts of interest.

## Supporting information



Supporting Information S1: Supplementary Methods S1–S5, Supplementary Results S1–S3, the supplementary figure legend, and reproducibility notes.
Supporting Information S3: Complete search strategies for PubMed/MEDLINE, Embase, Web of Science Core Collection, Scopus, and Wanfang Data.
Supporting Information S4: Completed PRISMA 2020 checklist.

Supporting Information S2: Supplementary Tables S1–S8, including excluded reports, scale harmonization, search yields, QUIPS judgments, robustness analyses, and evidence summaries.

Supporting Figure S1: Exploratory meta‐analysis of reperfusion‐treatment cohorts.

## Data Availability

The study‐level data and R code that support the findings of this study are available from the corresponding author upon reasonable request. Licensed database search exports are retained for audit and cannot be redistributed because of database access and licensing restrictions.
