# Supplementary material for: Early Fibrinogen‐to‐Albumin Ratio and Poor Functional Outcome After Acute Ischemic Stroke: A Systematic Review and Meta‐Analysis
Source: Brain Behav. 2026 Nov 13;16(11):e71929. doi: 10.1002/brb3.71929 (PMC13768740; doi:10.1002/brb3.71929)

Study	Participants	Weight(%)	Adjusted OR [95% CI]	P	I^2 (%)
Chen 2024	204	35.8	1.19 [1.03, 1.38]		
Tang 2024	376	16.7	1.47 [1.13, 1.91]		
Wei 2026	226	20.2	1.54 [1.22, 1.94]		
Ozdogru 2024	103	27.3	1.25 [1.04, 1.50]		
**Overall**	**909**	**100.0**	**1.32 [1.08, 1.60]**	**0.022**	**32.9**

Adjusted OR [95% CI]

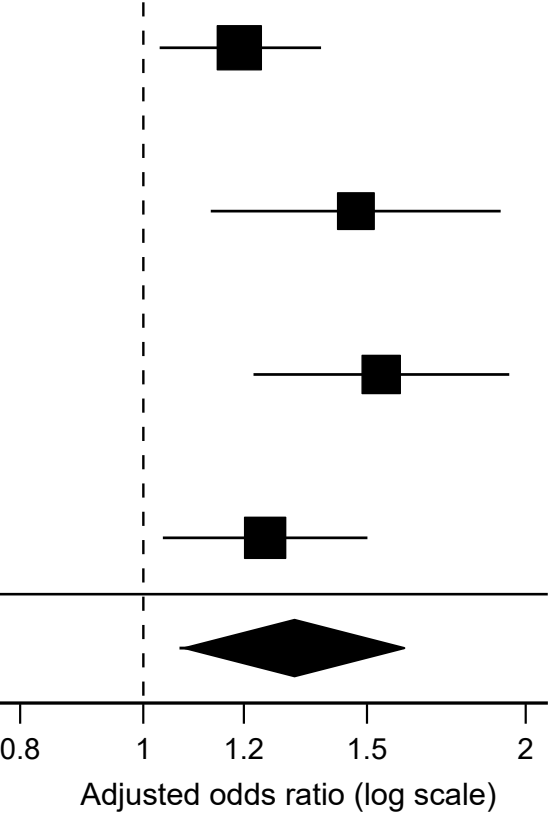

Supplement: Supplementary file 1 [file BRB3-16-e71929-s001.pdf]
