# Supplementary material for: Early Fibrinogen‐to‐Albumin Ratio and Poor Functional Outcome After Acute Ischemic Stroke: A Systematic Review and Meta‐Analysis
Source: Brain Behav. 2026 Nov 13;16(11):e71929. doi: 10.1002/brb3.71929 (PMC13768740; doi:10.1002/brb3.71929)
Supplement: Supplementary file 2 [file BRB3-16-e71929-s002.docx]

**Supplementary Table S1. Full-text reports excluded after eligibility assessment**

| **Study** | **Population or title cue** | **Primary reason for exclusion** |
| --- | --- | --- |
| Li 2023 | AIS after thrombectomy | Extractable effects used postoperative FAR and an unadjusted risk ratio; no eligible preprocedural effect was available |
| Zhang Zhonghua 2021 | Older adults with anterior-circulation large vessel occlusion after endovascular therapy | Sampling appeared to occur after treatment and the continuous effect scale could not be established |
| Tang 2023 | Successful recanalization after thrombectomy | FAR was measured after the procedure |
| Sun 2024 | AIS after intravenous thrombolysis | Outcome outside the review scope; no follow-up mRS was reported |
| Zhao 2024 | Recent small subcortical infarction | Outcome outside the review scope |
| Tang 2026 | AIS after intravenous thrombolysis | Outcome outside the review scope; no 90-day mRS was reported |
| Sayan 2023 | AIS diagnostic case-control cohort | Diagnostic design without an eligible adjusted prognostic effect |
| Shanghai NMF 2026 | Male patients with anterior-circulation large vessel occlusion after thrombectomy | Only a composite NLR x FAR effect was extractable |

Note. One principal exclusion reason is shown for each report.

**Supplementary Table S2. Harmonization of continuous FAR scales**

| **Study** | **Reported units and scale evidence** | **Original OR and regression increment** | **Conversion coefficient and formula** | **Harmonized effect** | **Meta-analysis decision** |
| --- | --- | --- | --- | --- | --- |
| Lai 2024 | Fibrinogen in mg/L and albumin in g/L; FAR approximately 96–124. These units imply Z=10 × FAR_100 for an unrescaled ratio. | OR 1.154 (1.006–1.323), treated as per 1-unit increase in reported FAR (Z). | c=10; one reported unit=0.1 FAR_100 unit. OR_100=1.154¹⁰; CI limits=1.006¹⁰ and 1.323¹⁰. | OR 4.188 (1.062-16.429) per 1-unit increase in FAR_100 | Included in the continuous meta-analysis |
| Zhang Yanyang 2024 | FAR approximately 83–126; concentration units not explicit. Z=10 × FAR_100 is inferred from these values. The original article states that continuous predictors entered the model at their original values (Section 2.2, p. 2392). | OR 1.484 (1.074–2.051) per 1-unit increase in reported FAR (Z), supported by the stated original-value entry; the mapping to FAR_100 remains inferred. | c=10; one reported unit=0.1 FAR_100 unit. OR_100=1.484¹⁰; CI limits=1.074¹⁰ and 2.051¹⁰. | OR 51.801 (2.042-1317.215) per 1-unit increase in FAR_100 | Included in the continuous meta-analysis |
| Ozdogru 2024 | Raw FAR approximately 0.082–0.094; regression variable expressed as FAR × 100 | OR 1.247 (1.036-1.501) per model unit | A 1-unit increase in FAR x 100 equals a 0.01 increase in raw FAR and a 1-unit increase in FAR_100 | OR 1.247 (1.036-1.501) per 1-unit increase in FAR_100 | Included in the continuous meta-analysis |

Note. X=FAR_100 and Z=the reported regression variable. For Z=cX, β_X=cβ_Z, SE_X=cSE_Z, and OR_X=OR_Zᶜ; confidence limits are transformed identically. Lai’s per-unit regression increment was assumed from the reported ratio. Zhang reported original-value entry of continuous predictors, but its c=10 measurement-scale relationship was inferred from FAR values. Neither conversion was author-confirmed. See Supplementary Methods S3 for the evidence and remaining uncertainties.

**Supplementary Table S3. Database search results**

| **Database** | **Records identified** | **Export and processing** |
| --- | --- | --- |
| PubMed/MEDLINE | 31 | Full XML export |
| Embase | 32 | Full RIS export |
| Web of Science Core Collection | 25 | Full RIS export |
| Scopus | 33 | Full RIS export |
| Wanfang | 57 | Main search screened page by page |
| Total | 178 | Four international bibliographic databases yielded 47 unique records after deduplication; 104 records were screened after addition of Wanfang. |

Note. The 57-record Wanfang main search was used in the PRISMA total; supplementary discovery sources were not added because of extensive overlap.

**Supplementary Table S4. QUIPS domain-level risk-of-bias judgments**

| **Study** | **Study participation** | **Study attrition** | **Prognostic factor measurement** | **Outcome measurement** | **Study confounding** | **Statistical analysis and reporting** |
| --- | --- | --- | --- | --- | --- | --- |
| Zheng 2021 | Low | Low | Low | Low | Low | Moderate |
| Zhai 2022 | Low | Low | Low | Low | Low | Low |
| Wang Y 2023 | Low | Low | Low | Low | Low | Low |
| Wang X 2024 | Low | Low | Low | Low | Low | Low |
| Chen 2024 | Low | Low | Low | Low | Low | Moderate |
| Tang 2024 | Low | Moderate | Low | Low | Low | Moderate |
| Lai 2024 | Low | Low | Low | Low | Moderate | Low |
| Ozdogru 2024 | Low | Low | Low | Low | Low | Low |
| Zhang Yanyang 2024 | Low | Low | Low | Low | Moderate | Moderate |
| Du 2026 | Moderate | High | Low | Low | Moderate | Moderate |
| Wei 2026 | Low | Low | Low | Low | Low | Low |
| Meng 2026 | Low | Low | Low | Low | Low | Moderate |

Note. Low, low risk of bias; Moderate, moderate risk of bias; High, high risk of bias. Entries are the final consensus judgments of two independent reviewers. No mechanical overall rating was assigned.

**Supplementary Table S5. Leave-one-out analyses**

| **Panel** | **Omitted study** | **Studies** | **Participants** | **Pooled OR (95% CI)** | **I² (%)** | **τ²** | **Q (df)** | **Q P value** |
| --- | --- | --- | --- | --- | --- | --- | --- | --- |
| Categorical FAR | Zheng 2021 | 4 | 10646 | 2.34 (1.13-4.83) | 87.0 | 0.142 | 23.16 (3) | <0.001 |
| Categorical FAR | Wang Y 2023 | 4 | 10159 | 2.18 (1.11-4.30) | 86.4 | 0.123 | 22.03 (3) | <0.001 |
| Categorical FAR | Wang X 2024 | 4 | 2222 | 2.87 (1.73-4.77) | 23.3 | 0.030 | 3.91 (3) | 0.271 |
| Categorical FAR | Meng 2026 | 4 | 10714 | 2.45 (1.27-4.72) | 86.7 | 0.118 | 22.48 (3) | <0.001 |
| Categorical FAR | Du 2026 | 4 | 10131 | 1.88 (1.15-3.07) | 20.5 | 0.029 | 3.77 (3) | 0.287 |
| Continuous FAR_100 | Zhai 2022 | 6 | 1347 | 1.34 (1.05-1.71) | 59.2 | 0.007 | 12.25 (5) | 0.031 |
| Continuous FAR_100 | Chen 2024 | 6 | 1407 | 1.34 (1.14-1.58) | 51.7 | <0.001 | 10.35 (5) | 0.066 |
| Continuous FAR_100 | Tang 2024 | 6 | 1235 | 1.29 (1.11-1.50) | 56.0 | <0.001 | 11.37 (5) | 0.044 |
| Continuous FAR_100 | Lai 2024 | 6 | 1415 | 1.30 (1.13-1.48) | 47.2 | <0.001 | 9.46 (5) | 0.092 |
| Continuous FAR_100 | Wei 2026 | 6 | 1385 | 1.28 (1.10-1.48) | 50.1 | <0.001 | 10.03 (5) | 0.074 |
| Continuous FAR_100 | Zhang Yanyang 2024 | 6 | 1369 | 1.30 (1.16-1.46) | 31.4 | <0.001 | 7.29 (5) | 0.200 |
| Continuous FAR_100 | Ozdogru 2024 | 6 | 1508 | 1.34 (1.09-1.64) | 58.4 | 0.005 | 12.03 (5) | 0.034 |

Note. Each row shows the pooled estimate after omission of the named study. All analyses used REML random effects and modified Hartung-Knapp confidence intervals.

**Supplementary Table S6. Scale-restriction and exploratory reperfusion-treatment analyses**

| **Analysis** | **Studies** | **Participants** | **Effect measure** | **Estimate (95% CI)** | **I² (%)** | **P value for heterogeneity/ interaction** | **Interpretation** |
| --- | --- | --- | --- | --- | --- | --- | --- |
| No mathematical rescaling required (excludes Lai 2024 and Zhang Yanyang 2024) | 5 | 1173 | OR per 1-unit increase in FAR_100 | 1.29 (1.16-1.45) | 10.5 | 0.346 | Scale robustness analysis |
| Explicit or confirmed FAR_100 definition (also excludes Tang 2024) | 4 | 797 | OR per 1-unit increase in FAR_100 | 1.28 (1.12-1.47) | 14.0 | 0.322 | Scale robustness analysis |
| Restricted to reperfusion-treatment cohorts | 4 | 909 | OR per 1-unit increase in FAR_100 | 1.32 (1.08-1.60) | 32.9 | 0.215 | Exploratory restricted analysis |
| Reperfusion-treatment setting interaction | 7 | 1,611 | Ratio of odds ratios | 1.02 (0.65-1.59) | Not applicable | 0.920 | Exploratory study-level moderator test |

Note. The treatment-setting interaction is exploratory because only seven continuous-effect studies were available.

**Supplementary Table S7. Full statistical details of primary and supporting meta-analyses**

| **Analysis** | **Exposure contrast** | **Studies** | **Participants** | **Pooled OR (95% CI)** | **I² (%)** | **τ²** | **Q (df)** | **Q P value** | **95% prediction interval** | **Role** |
| --- | --- | --- | --- | --- | --- | --- | --- | --- | --- | --- |
| Primary categorical FAR | Study-defined high or highest category vs low or lowest category | 5 | 10968 | 2.32 (1.37-3.91) | 82.7 | 0.108 | 23.16 (4) | <0.001 | 0.69-7.74 | Primary analysis |
| Primary continuous FAR | Per 1-unit increase in FAR_100 | 7 | 1611 | 1.30 (1.14-1.48) | 51.1 | <0.001 | 12.27 (6) | 0.056 | 1.13-1.50 | Primary analysis |
| No mathematical rescaling required (excludes Lai 2024 and Zhang Yanyang 2024) | Per 1-unit increase in FAR_100 | 5 | 1173 | 1.29 (1.16-1.45) | 10.5 | <0.001 | 4.47 (4) | 0.346 | Not calculated | Scale robustness analysis |
| Explicit or confirmed FAR_100 definition (also excludes Tang 2024) | Per 1-unit increase in FAR_100 | 4 | 797 | 1.28 (1.12-1.47) | 14.0 | <0.001 | 3.49 (3) | 0.322 | Not calculated | Scale robustness analysis |
| Restricted to reperfusion-treatment cohorts | Per 1-unit increase in FAR_100 | 4 | 909 | 1.32 (1.08-1.60) | 32.9 | 0.005 | 4.47 (3) | 0.215 | Not calculated | Exploratory restricted analysis |

Note. All pooled estimates used REML random effects and modified Hartung-Knapp 95% confidence intervals. τ² was estimated by REML on the squared log-OR scale. I²=max{0, [Q−(k−1)]/Q} × 100%; Q P values are from Cochran’s Q test against a chi-square distribution with k−1 degrees of freedom. Categorical and continuous FAR are separate estimands. A concise summary is presented in Table 2 of the main manuscript.

**Supplementary Table S8. Interpretive summary of the evidence**

| **Finding** | **Evidence** | **Interpretation** | **Implication** |
| --- | --- | --- | --- |
| Categorical FAR | 5 studies; 10,968 participants; pooled OR 2.32 (95% CI 1.37-3.91) | Study-defined higher FAR categories were associated with more than twice the odds of poor functional outcome. | Study-defined higher FAR categories were associated with higher observed odds of poor outcome, although a universal cutoff was not estimated. |
| Continuous FAR_100 | 7 studies; 1,611 participants; OR 1.30 (95% CI 1.14-1.48) per 1-unit increase; 95% PI 1.13-1.50 | The harmonized continuous association and prediction interval supported a positive association across the settings represented. | Future prognostic models should retain FAR as a continuous variable and assess its incremental predictive value. |
| Robustness | All leave-one-out CIs remained above 1; no-rescaling OR 1.29 (95% CI 1.16-1.45); explicit or confirmed FAR_100 OR 1.28 (95% CI 1.12-1.47) | Neither an individual study nor mathematical scale conversion explained the continuous association. | Standardized reporting of assay units, sampling windows and FAR_100 would make estimates directly comparable. |
| Reperfusion-treatment cohorts | 4 studies; 909 participants; OR 1.32 (95% CI 1.08-1.60) per 1-unit increase | The association was also present in cohorts treated with thrombolysis or endovascular therapy. | FAR merits prospective evaluation as a baseline adjunct in time-sensitive reperfusion pathways. |

Note. The statements summarize prognostic associations and do not imply a treatment effect.
