# Supplementary material for: Early Fibrinogen‐to‐Albumin Ratio and Poor Functional Outcome After Acute Ischemic Stroke: A Systematic Review and Meta‐Analysis
Source: Brain Behav. 2026 Nov 13;16(11):e71929. doi: 10.1002/brb3.71929 (PMC13768740; doi:10.1002/brb3.71929)
Supplement: Supplementary file 3 [file BRB3-16-e71929-s003.docx]

**Supplementary Information**

# Early Fibrinogen-to-Albumin Ratio and Poor Functional Outcome After Acute Ischemic Stroke: A Systematic Review and Meta-analysis

# Contents

- Supplementary Methods S1. Protocol, eligibility, and effect selection
- Supplementary Methods S2. Study identification, deduplication, selection, and data extraction
- Supplementary Methods S3. Continuous-scale harmonization
- Supplementary Methods S4. Risk-of-bias assessment
- Supplementary Methods S5. Statistical methods
- Supplementary Results S1. Leave-one-out analyses
- Supplementary Results S2. Continuous-scale robustness analyses
- Supplementary Results S3. Reperfusion-treatment cohorts
- Supplementary Table S1. Full-text reports excluded after eligibility assessment
- Supplementary Table S2. Harmonization of continuous FAR scales
- Supplementary Table S3. Database search results
- Supplementary Table S4. QUIPS domain-level risk-of-bias judgments
- Supplementary Table S5. Leave-one-out analyses
- Supplementary Table S6. Scale-restriction and exploratory reperfusion-treatment analyses
- Supplementary Table S7. Full statistical details of primary and supporting meta-analyses
- Supplementary Table S8. Interpretive summary of the evidence
- Supplementary Figure S1. Reperfusion-treatment cohort analysis
- Supplementary Note S1. Data and code availability
- Supplementary Note S2. Protocol registration and amendments

# **Supplementary Methods S1. Protocol, eligibility, and effect selection**

The review followed PRISMA 2020 and MOOSE recommendations and was registered in PROSPERO (CRD420261459955). Eligible designs were prospective or retrospective cohorts, secondary analyses of registries, and case-control studies that allowed extraction of a prognostic association.

Reports limited to mortality, early neurological deterioration, hemorrhagic transformation, or diagnostic performance were excluded unless they also reported an eligible adjusted association with follow-up functional outcome. Within each cohort and analysis panel, we selected the prespecified primary time point and the most fully adjusted eligible model. Categorical and continuous effects were treated as separate exposure contrasts.

Adults aged 18 years or older with acute ischemic stroke confirmed by computed tomography or magnetic resonance imaging were eligible. Unselected acute ischemic stroke cohorts, intravenous thrombolysis cohorts, endovascular treatment cohorts, and cohorts restricted to a specific ischemic stroke subtype were included. Mixed populations were eligible only when acute ischemic stroke data could be extracted separately.

Early FAR was defined as the first available value obtained at admission or baseline, on the first morning after admission when sampling was not known to follow treatment, or before intravenous thrombolysis, endovascular treatment, or surgery. Studies reporting only post-treatment FAR were excluded. FAR had to be extractable as an independent exposure; composite indices without a FAR-specific association were not eligible for quantitative synthesis.

The outcome was poor functional outcome assessed between 30 days and one year with a dichotomized modified Rankin Scale (mRS). Eligible definitions included mRS 3 to 6, mRS greater than 2, mRS 4 to 6, or another threshold clearly representing death or functional dependence. When several follow-up points were reported, the study-defined primary functional outcome was selected. If no primary time point was identified, 90 days or three months was preferred.

The categorical panel used an adjusted odds ratio comparing a study-defined higher FAR group with a lower group, or the highest with the lowest quantile. The continuous panel used an adjusted odds ratio for poor functional outcome per 1-unit increase in FAR_100. One estimate from each cohort was retained within each panel. Chen 2024 reported a categorical reverse model in which poor functional outcome predicted high FAR status; that estimate did not match the prognostic estimand and was not pooled. Its eligible continuous prognostic model was included. Ozdogru 2024 modeled FAR multiplied by 100 and was directly compatible with FAR_100.

# **Supplementary Methods S2. Study identification, deduplication, selection, and data extraction**

PubMed/MEDLINE, Embase, Web of Science Core Collection, Scopus, and Wanfang were searched from inception through July 23, 2026. No prognosis, outcome, or mRS term block was required. Europe PMC and OpenAlex supported supplementary discovery and full-text location; Crossref supported bibliographic verification. Reference lists and forward citations were screened. The China National Knowledge Infrastructure was not searched. Full queries are provided in the Supplementary Search Strategies file.

PubMed/MEDLINE, Embase, Scopus, and Web of Science contributed 121 exported records. DOI was used as the primary deduplication key, followed by normalized title and first author. Deduplication removed 74 records and left 47 unique records from the four international bibliographic databases. After the 57 Wanfang main-search records were added, 104 records underwent title and abstract screening.

Two reviewers independently screened titles and abstracts, assessed full texts, and extracted data. Extracted items included design, population, sample size, FAR sampling time and formula, exposure contrast, follow-up, mRS threshold, event count, effect estimate and confidence interval, adjustment variables, and attrition information. Disagreements were resolved by returning to the source report and discussion.

Complete formal database strings are supplied in the separate Supplementary Search Strategies file.

# Supplementary Methods S3. Continuous-scale harmonization

The common continuous scale used g/L for both fibrinogen and albumin:

FAR_100 = 100 × fibrinogen (g/L) / albumin (g/L)

Let X denote FAR_100 and Z the reported regression variable. If Z=cX and the adjusted OR is per 1-unit increase in Z, then 1 unit of Z equals 1/c units of X. Rewriting the model logit(p)=α+β_Z × Z as logit(p)=α+(cβ_Z) × X gives:

β_X = cβ_Z; SE_X = cSE_Z; OR_X = OR_Zᶜ; lower CI_X = lower CI_Zᶜ; upper CI_X = upper CI_Zᶜ (c>0).

Thus c is the number of reported-scale units corresponding to a 1-unit increase in FAR_100; standard errors are multiplied by the same positive factor. Fibrinogen (mg/L)/albumin (g/L)=1,000 × fibrinogen (g/L)/albumin (g/L)=10 × FAR_100. Under this definition, c=10, one reported unit equals 0.1 FAR_100 unit, and the OR and both confidence limits are raised to the 10th power.

Lai 2024 reported fibrinogen in mg/L and albumin in g/L, supporting Z=10 × FAR_100 for the unrescaled ratio; its OR was treated as per 1-unit increase in that ratio. Zhang Yanyang 2024 stated that continuous variables, including FAR, were entered at their original values (original article, Section 2.2, p. 2392), supporting an OR per 1-unit increase in reported FAR. Its FAR values of approximately 83 to 126 were compatible with the same tenfold relationship, although the underlying concentration units were not specified. Thus, c=10 was inferred from Lai’s laboratory units and Zhang’s reported FAR values. The authors of Lai (2024) and Zhang Yanyang (2024) were not contacted to verify the regression increments. For Lai, laboratory units alone do not exclude additional rescaling, standardization, or log transformation before regression. For Zhang, the remaining uncertainty concerns the FAR unit convention despite the stated use of original values. Lai’s OR 1.154 (95% CI 1.006–1.323) becomes 4.188 (1.062–16.429), and Zhang’s OR 1.484 (1.074–2.051) becomes 51.801 (2.042–1317.215) per 1-unit increase in FAR_100. Analyses excluding both studies are reported separately (Figure 4C; Supplementary Table S6). Ozdogru 2024 used FAR multiplied by 100, so no numerical conversion was required. Tang 2024 reported values compatible with FAR_100, but the definition was not as explicit as in the strict scale subset.

# **Supplementary Methods S4. Risk-of-bias assessment**

Two reviewers independently assessed study participation, study attrition, prognostic factor measurement, outcome measurement, study confounding, and statistical analysis and reporting with the Quality In Prognosis Studies tool. Domains were rated low, moderate, or high risk after considering the likely direction and effect of each concern. Disagreements were resolved by checking the source report and reaching consensus; no overall rating was assigned mechanically from the highest single-domain judgment.

# **Supplementary Methods S5. Statistical methods**

Adjusted odds ratios were analyzed on the log scale. Standard errors were calculated as SE=[ln(upper 95% CI)−ln(lower 95% CI)]/(2×1.96). Between-study variance (τ²), on the squared log-OR scale, was estimated by restricted maximum likelihood. Confidence intervals used the modified Hartung-Knapp method; when the ordinary Hartung-Knapp variance multiplier was below 1, it was set to 1. Pooled-effect confidence intervals and tests used a t distribution with k−1 degrees of freedom. Prediction intervals used the modified variance and a t distribution with k−2 degrees of freedom.

Categorical and continuous FAR were analyzed separately. Leave-one-out analyses sequentially omitted one study from each primary panel. Continuous-scale robustness was examined by first excluding the two studies that required mathematical conversion and then retaining only studies with an explicit or confirmed FAR_100 definition. A separate exploratory analysis was restricted to cohorts explicitly treated with intravenous thrombolysis or endovascular therapy. A study-level mixed effects meta-regression tested interaction by reperfusion-treatment setting.

Funnel plots and Egger's tests were not used because each primary effect pool contained fewer than 10 studies. Analyses and all statistical figures were produced in R 4.6.1, principally with metafor 5.0-1.

Cochran’s Q was calculated as Q=Σw_i(y_i−ȳ_FE)², where y_i=ln(OR_i), w_i=1/SE_i², and ȳ_FE=Σw_i y_i/Σw_i. Its P value was obtained from the upper tail of a chi-square distribution with k−1 degrees of freedom. I² was calculated as max{0, [Q−(k−1)]/Q} × 100%, rather than from the REML estimate of τ². Values of Q, its degrees of freedom and P value, I², and τ² are reported together in Supplementary Table S7; heterogeneity P values are distinct from pooled-effect P values. Prediction intervals were calculated only for the two primary analyses. The exploratory mixed-effects meta-regression used REML estimation with a Knapp-Hartung F test and reported the ratio of ORs, its 95% CI, and the interaction P value. Effect and interaction tests were two-sided with α=0.05. Participant-level missing values were not imputed and unadjusted effects were not reconstructed from 2-by-2 tables.

Forest plots placed structured numerical tables beside study-specific and pooled estimates.

# Supplementary Results S1. Leave-one-out analyses

For categorical FAR, pooled odds ratios after sequential study omission ranged from 1.88 to 2.87. Every recalculated 95% confidence interval remained above 1, with the lowest lower limit equal to 1.11. I² ranged from 20.5% to 87.0%. Omission of Wang X 2024 reduced I² to 23.3%, and omission of Du 2026 reduced it to 20.5%. The positive pooled association was retained in both cases. Detailed estimates are shown in Figure 4A and Supplementary Table S5.

For continuous FAR_100, leave-one-out pooled odds ratios ranged from 1.28 to 1.34 per 1-unit increase. Every recalculated 95% confidence interval remained above 1, with the lowest lower limit equal to 1.05. I² ranged from 31.4% to 59.2%. Detailed estimates are shown in Figure 4B and Supplementary Table S5.

# Supplementary Results S2. Continuous-scale robustness analyses

After Lai 2024 and Zhang Yanyang 2024 were removed, five studies with 1,173 participants produced an odds ratio of 1.29 per 1-unit increase in FAR_100 (95% confidence interval 1.16 to 1.45). I² fell to 10.5%, and Q was 4.47 with 4 degrees of freedom (P=0.346). This restriction is shown in Figure 4C and Supplementary Table S6.

The stricter analysis retained Zhai 2022, Chen 2024, Wei 2026, and Ozdogru 2024. These four studies included 797 participants and produced an odds ratio of 1.28 (95% confidence interval 1.12 to 1.47). I² was 14.0%, and Q was 3.49 with 3 degrees of freedom (P=0.322). The similarity of these estimates to the seven-study primary result shows that mathematical rescaling did not explain the continuous association. This restriction is shown in Figure 4C and Supplementary Table S6.

# Supplementary Results S3. Reperfusion-treatment cohorts

Four studies explicitly enrolled patients treated with intravenous thrombolysis or endovascular therapy. The analysis included 909 participants. Each 1-unit increase in FAR_100 was associated with poor functional outcome (odds ratio 1.32, 95% confidence interval 1.08 to 1.60; τ²=0.0054; I²=32.9%; Q=4.47, 3 degrees of freedom; P=0.215). Study-specific estimates are shown in Supplementary Figure S1 and Supplementary Table S6.

The exploratory meta-regression produced a ratio of odds ratios of 1.02 for other or unselected acute ischemic stroke cohorts versus explicit reperfusion-treatment cohorts (95% confidence interval 0.65 to 1.59; F[1,5]=0.011; P for interaction=0.920). The test was underpowered and does not establish equivalence between treatment settings.

# Supplementary tables

Tables 1 and 2 are provided in the main manuscript; Supplementary Tables S1-S8 are provided in the separate Supplementary Tables file. The statistical tables report the underlying study-level and pooled estimates.

# Supplementary figure legend

Supplementary Figure S1. Exploratory analysis restricted to reperfusion-treatment cohorts. The left table reports study sample sizes, adjusted odds ratios with 95% confidence intervals, random-effects weights, the pooled-effect P value, and I². The P value is from a two-sided modified Hartung-Knapp test of the pooled null OR=1; I² is Q-based. Squares and horizontal lines in the forest plot show study-specific estimates and confidence intervals; square size reflects the random-effects weight. The diamond shows the restricted maximum likelihood random-effects estimate with a modified Hartung-Knapp 95% confidence interval. CI indicates confidence interval; FAR, fibrinogen-to-albumin ratio; OR, odds ratio. Effects are reported per 1-unit increase in FAR_100, defined as fibrinogen (g/L) divided by albumin (g/L) and multiplied by 100.

# Supplementary Note S1. Data and code availability

The study-level effect data, meta-analysis summaries, and R code used for the analyses and figures are available from the corresponding author upon reasonable request. Full-text exclusions are reported in Supplementary Table S1, and domain-level QUIPS judgments are reported in Supplementary Table S4. Licensed database search exports are retained for audit and cannot be redistributed because of database access and licensing restrictions.

**Supplementary Note S2. Protocol registration and amendments**

The review was registered in PROSPERO (CRD420261459955). Protocol version 1.1 added leave-one-out analyses, two nested continuous-scale restrictions, and analyses of reperfusion-treatment cohorts as robustness or exploratory analyses. These additions did not change the review question, eligibility criteria, the categorical and continuous primary analyses, or the primary statistical models. The protocol is available from the corresponding author upon reasonable request.
